# Decreased cholinesterase level combined with renal dysfunction and sympathetic denervation associated with increased cardiac mortality in systolic heart failure

**DOI:** 10.3389/fcvm.2023.1131282

**Published:** 2023-09-29

**Authors:** Takahiro Doi, Tomoaki Nakata, Taro Tsuzuki, Tomohiro Mita, Daigo Nagahara, Satoshi Yuda, Akiyoshi Hashimoto

**Affiliations:** ^1^ Department of Cardiology, Teine Keijinkai Hospital, Sapporo, Japan; ^2^ Department of Cardiology, Hakodate Goryokaku Hospital, Hakodate, Japan; ^3^ Department of Cardiology, Renal and Metabolic Medicine, Sapporo Medical University, Sapporo, Japan

**Keywords:** heart failure, prognosis, malnutrition, serum cholinesterase, risk stratification

## Abstract

**Aims:**

Cardiac mortality in patients with heart failure (HF) is likely to be aggravated by malnutrition, assessed by serum cholinesterase (ChE) level, as well as by kidney dysfunction or impairment of cardiac sympathetic denervation. Their prognostic interactions, however, have not been determined.

**Methods:**

A total of 991 systolic HF patients were enrolled in our HF database following clinical evaluation including evaluation of the nutrition state and assessment of standardized heart-to-mediastinum ratio (sHMR) of iodine-123-labeled meta-iodobenzylguanidine activity. Patients were followed up for an average of 43 months with the primary endpoint of fatal cardiac events (CEs).

**Results:**

The CE patient group had a lower level of ChE, lower estimated glomerular filtration rate (eGFR) and lower late sHMR than those in the non-CE patient group. A five-parameter model with the addition of serum ChE selected in the multivariate logistic analysis (model 2) significantly increased the AUC predicting risk of cardiac events compared with a four-parameter model without serum ChE (model 1), and net reclassification analysis also suggested that the model with the addition of serum cholinesterase significantly improved cardiac event prediction. Moreover, in overall multivariate Cox hazard analysis, serum ChE, eGFR and late sHMR were identified to be significant prognostic determinants. HF patients with two or all of the prognostic variables of serum ChE < 230 U/L, eGFR < 48.8 ml/min/1.73 m^2^ and late sHMR < 1.90 had significantly and incrementally increased CE rates compared to those in HF patients with none or only one of the prognostic variables.

**Conclusion:**

Decreases in cholinesterase level and kidney function further increase cardiac mortality risk in HF patients with impairment of cardiac sympathetic innervation.

## Introduction

Obesity is well known to be an independent risk factor for metabolic, endocrine and cardiovascular diseases (1). On the other hand, it has been shown that heart failure (HF) patients who are malnourished or experiencing rapid weight loss may have an increased prognostic risk independent of age, New York Heart Association (NYHA) functional class, or cardiac functions (2–4). These findings clearly demonstrate the importance of controlling energy intake and body weight for better prognosis, as indicated by many prophylactic or treatment guidelines for atherosclerotic disorders. The dichotomic aspects have emphasized the necessity to appropriately select a nutritional intervention strategy in association with the nutrition state, patient activity, baseline disease, comorbidities and cardiac function. Although body mass index (BMI) is conventionally used for the assessment of obesity or calorie intake in relatively heathy subjects, the usefulness of BMI may be limited for identifying a malnutrition state or sub-clinical cachexia in patients with pathologic conditions. This is because a pathological undernutrition state is related to many factors such as protein catabolism, lipolysis, bone loss, kidney dysfunction and anemia together with increases in sympathetic nerve activity, inflammatory cytokines and/or insulin resistance (5). It has recently been shown that serum cholinesterase can be an indicator of hepatic function and nutrition, and several investigations have revealed its close correlation with prognosis in HF patients (6, 7). Although these biomarkers are speculated to increase cardiac mortality by the development of a vicious cycle, there are few reports on the prognostic interactions in HF patients.

With reference to previous studies on the prognosis of HF (8–13), the aim of the present study was to determine prognostic incremental values for risk stratification of HF patients with reduced left ventricular ejection fraction (LVEF), with particular focus on the prognostic interactions of serum cholinesterase levels, renal function and cardiac sympathetic innervation as nutritional parameters.

## Materials and methods

### Study design and study subjects

A total of 991 consecutive patients with symptomatic HF and echocardiographic LVEF <50% who were admitted to our hospital between April 2010 and December 2016 and underwent myocardial scintigraphy of ^123^I-labeled meta-iodobenzylguanidine (MIBG) for prognostic evaluation after compensatory and optimal medical therapy were retrospectively enrolled in our HF database.

The inclusion criteria for this retrospective study were symptomatic HF requiring hospitalization, LVEF <50% determined by echocardiography, and age >20 years. Patients who refused resuscitation, patients who had obvious malignancy or hemorrhagic disease, and patients who were younger than 20 years of age were excluded. The patients included 738 males (74.5%). The mean age of the patients was 67.5 ± 12.9 years and the mean LVEF was 32.7 ± 11.3%. The diagnosis of HF was made by the Framingham criteria including typical symptoms of jugular venous distention, peripheral edema, pulmonary rales, S3 or S4 gallop sounds, and tachycardia. A chest x-ray was taken and two-dimensional echocardiography was performed to corroborate the diagnosis and rule out other diseases with similar symptoms and signs. In addition to a history of myocardial infarction or coronary revascularization, the etiology of HF, such as ischemic or nonischemic, was differentiated by electrocardiography, echocardiography, scintigraphy, or a combination of these when necessary as well as by coronary vascular information obtained by computed tomography, magnetic resonance imaging, and invasive selective coronary angiography. Just before discharge, blood tests were performed to measure the levels of hemoglobin (Hb), albumin, lipid profiles, glutamic-oxaloacetic transferase (GOT), glutamic-pyruvic transferase (GPT), lactate dehydrogenase (LDH), serum ChE, creatinine and brain natriuretic peptide (BNP). Renal function was evaluated by estimated glomerular filtration rate (eGFR) using the standard formula. The baseline geriatric nutrition risk index (GNRI) was calculated from serum albumin and BMI using the following formula: GNRI = 14.89 × serum albumin (g/dl) + 41.7 × present body weight/[height^2^ (m^2^) × 22] = 14.89 × serum albumin (g/dl) + 41.7 × BMI/22. Plasma BNP levels were measured in 598 patients (60.3%) and NT-pro BNP levels were measured in the remaining 393 patients (39.6%) before discharge when heart failure was stable. To evaluate BNP and NT-pro BNP in an integrated manner, all of the HF patients were classified into four groups according to the following values based on ESC HF guidelines: 0–40 pg/ml and 0–125 pg/ml for stage 1, 41–100 pg/ml and 126–400 pg/ml for stage 2, 101–200 pg/ml and 401–900 pg/ml for stage 3, and 201 pg/ml and 901 pg/ml for stage 4, respectively.

### Echocardiographic assessment

Two-dimensional echocardiography was performed by echocardiographic technicians with no knowledge of the patients' clinical data in the left lateral recumbent position using a commercially available ultrasound machine equipped with a 2.5 MHz variable frequency transducer to measure the parameters from views of the paracentral long axis and short axis and apical 4, 3, and 2 chambers. The following echocardiographic parameters were measured at the time of compensated heart failure prior to discharge: left atrium diameter (LAD; mm), left ventricular end-diastolic diameter (LVDd; mm), LVEF (%) calculated using the biplane modified Simpson's method, left ventricular volume at end-diastole (EDV; ml), left ventricular volume at end-systole (ESV; ml) and septal *E*/*e*’ (14, 15).

### Measurement of sympathetic denervation of the left ventricle

Cardiac sympathetic innervation by ^123^I-labeled MIBG of 111 MBq was quantitatively assessed in patients with compensated heart failure in a fasting and resting state by cardiac imaging using a gamma camera with a low-energy generic collimator at 15–30 min (early images) and 4 h (late images) after an intravenous tracer injection, as previously described (9–11).

Cardiac ^123^I-MIBG activity was measured as heart-to-mediastinum ratio (HMR) using dedicated MIBG software (*Smart MIBG Software, Tokyo, Japan*) operated by experienced nuclear medicine technicians with no knowledge of the patients' clinical data, automatically setting the region of interest to the upper mediastinum and the entire heart on a planar anterior image. ^123^I-MIBG washout kinetics from the left ventricle were calculated from early and late cardiac ^123^I-MIBG activities as washout rate (WR). The HMR of cardiac ^123^I-MIBG was measured using a late image and standardized as sHMR for medium energy collimator conditions by a mathematical method established in a cross-calibration phantom experiment (12, 16). This method not only minimizes the variability of ^123^I-MIBG HMR but also makes it possible to compare HMR data from sHMR regardless of differences in data acquisition methods such as data obtained using low- and medium-energy collimators or data obtained at different research facilities.

### Follow-up protocol

HF patients enrolled in our hospital HF database were regularly followed up by experienced cardiologists in the out-patient clinic for one year for a longer period if the patients survived. The cardiac events (CEs) that were considered as the primary endpoints of the present study were as follows: fatal CEs such as sudden cardiac death, death due to refractory and progressive pump failure, fatal sustained ventricular tachyarrhythmias, including ventricular tachycardia and ventricular fibrillation, and appropriate implantable cardioverter defibrillator (ICD) therapy against fatal ventricular arrhythmias. Clinical findings were confirmed by reviewing the patients' medical records and outcome analysis was retrospectively performed. In HF patients enrolled in the present study, sudden cardiac death was defined as witnessed cardiac arrest and death within one hour of acute onset or unexpected death in patients who had survived for the previous 24 h. The present study was conducted in accordance with the principles outlined in the Declaration of Helsinki and was approved by our institutional ethics committee for enrollment in our database and use of data for clinical research. The present study was approved by the Ethics Committee of Obihiro Kosei Hospital. Its approval number was 2016-015. Due to the retrospective, non-interventional, observational nature of the study as well as the fact that the clinical research was publicly disclosed on the institution's Web site and the fact that comprehensive consent was determined by the institution's ethics committee, the need for each patient's written informed consent was waived.

### Statistical analysis

Statistical values are presented as means ± SD. Means of values in the two groups were compared by the unpaired *t*-test and categorical variables were compared by the *χ*^2^ test. New parameters were added to the conventional risk factors for patients with HF, and area under the curve (AUC) and net reclassification index (NRI) were used to assess whether the prognostic evaluation was significantly improved. Reclassification was additionally used to re-evaluate the results.

A time-dependent cumulative event-free curve was generated by Kaplan-Meier analysis using the key parameters identified in the present study, and log-rank tests were also used to compare the curves as needed. The number of significant variables identified in the univariate analysis was statistically appropriate for the number of cardiac events in the present study, and multivariate analysis with the Cox proportional hazards model was performed to calculate hazard ratios and 95% confidence intervals (CIs) for the significant variables. Receiver operating characteristic (ROC) analysis was performed to determine the optimal cut-off values for the independent significant parameters of CEs. Statistical analysis in the present study was performed using the computer software SAS for Windows (version 9.4, SAS Institute, Cary, North Carolina, USA) and Mathematica software for Windows (version 12.3, Wolfram Research Inc., Champaign, IL. USA). *P* values less than 0.05 were considered significant.

## Results

**Figure 1:**
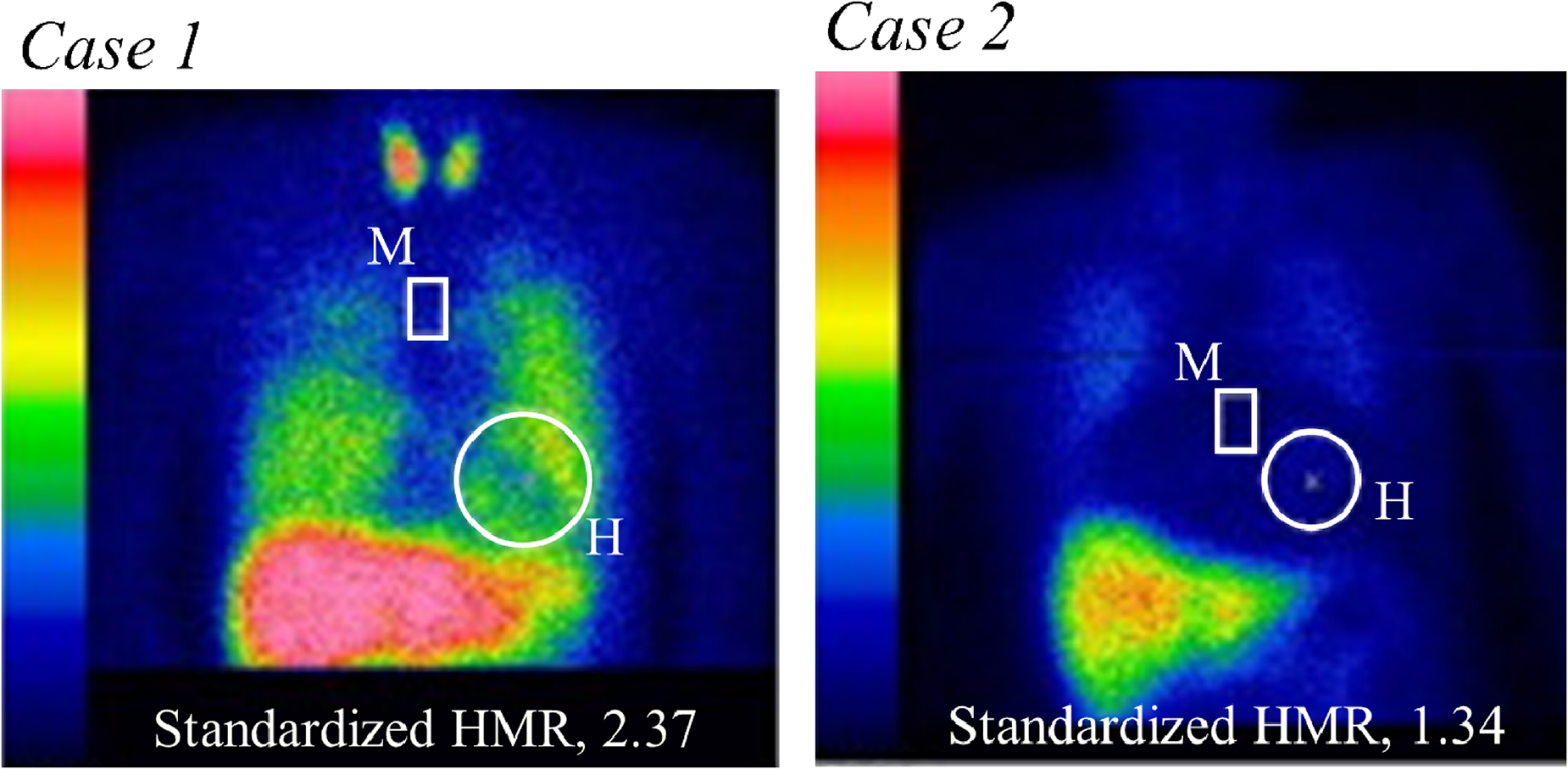
Measurements of heart (H)-to-mediastinum (M) ratio (HMR) of ^123^I-MIBG activity using a late planar anterior image standardized with dedicated MIBG software (*smart MIBG software, Tokyo, Japan*). Case 1 (*left panel*): In a 51-year-old male, LVEF was reduced (21%) due to ischemic cardiomyopathy, but eGFR (67 ml/min/1.73 m^2^), serum cholinesterase (330 U/L) and late standardized HMR (2.37) were nearly normal. No fatal cardiac events occurred during the observation period. Case 2 (*right panel*): A 71-year-old woman underwent cardiac resynchronization therapy after refractory heart failure management with optimal medical therapy alone. She had significantly decreased values of LVEF (28%) due to non-ischemic cardiomyopathy, eGFR (23 ml/min/1.73 m^2^), serum cholinesterase (136 U/L), and late standardized HMR (1.34). The patient died of progressive pump failure during the observation period.

Following measurement of serum ChE and calculation of eGFR, cardiac sympathetic innervation was quantified as standardized HMR of MIBG activity as shown by two typical cases (Figure 1). Despite having almost identical levels of cardiac dysfunction, Case 1 with almost normal values of serum ChE, eGFR and late sHMR (2.37) had no CEs, while Case 2 with apparent decreases in serum ChE, eGFR and late sHMR (1.34) died of progressive pump failure during the observation period. In 313 HF patients (31.5%), CEs recorded during a mean follow-up period of 43 months were as follows: sudden cardiac death in 29 patients, cardiac death due to refractory and progressive pump failure in 240 patients, lethal ventricular tachyarrhythmias in 22 patients and appropriate ICD treatment against lethal ventricular tachyarrhythmias in 22 patients.

HF patients in the CE group were older than those in the non-CE group, and HF patients in the CE group had a greater NYHA functional class, lower eGFR (36.7 ± 25.3 vs. 50.1 ± 27.8 ml/min/1.73 m^2^, *P *< 0.0001), lower BMI (22.1 ± 4.3 vs. 23.0 ± 4.5, *P *= 0.0027) and lower serum ChE (216.3 ± 82.5 vs. 258.5 ± 84.6 U/L, *P *< 0.0001) than those in patients in the group without CEs (Table 1). Patients in the CE group had a significantly lower late sHMR than that in patients in the non-CE group: 1.72 ± 0.41 vs. 2.00 ± 0.48, *P *< 0.0001 (Table 2).

**Table 1:** Comparison of clinical data for patients with and those without cardiac events.

	Cardiac event group (*n* = 313)	Non-cardiac event group (*n* = 678)	*P*-value
Age (years)	71.1 ± 11.1	65.7 ± 13.3	*P* < 0.0001
Gender (male/female)	241/72	497/181	*P* = 0.1982
NYHA (Ⅰ/Ⅱ/Ⅲ/Ⅳ)	138/85/76/14	620/45/7/6	*P* < 0.0001
BMI (kg/m^2^)	22.1 ± 4.3	23.0 ± 4.5	*P* = 0.0027
Past history			
Hypertension	148 (47.2%)	334 (49.3%)	ns
Diabetes mellitus	112 (35.7%)	250 (36.8%)	ns
Dyslipidemia	119 (38.0%)	292 (43.1%)	ns
Atrial fibrillation	114 (36.4%)	216 (31.9%)	ns
Ventricular tachycardia/Ventricular fibrillation	84 (26.8%)	100 (14.7%)	*P* < 0.0001
Hemodialysis	62 (19.8%)	69 (10.1%)	*P* < 0.0001
Etiology			
Ischemic	159 (50.7%)	300 (44.2%)	ns
Device implantation			
ICD implantation	60 (19.1%)	81 (11.9%)	*P* = 0.0028
CRT implantation	37 (11.8%)	68 (10.0%)	ns
Laboratory data			
Hemoglobin (g/dl)	11.6 ± 2.1	12.5 ± 2.3	*P* < 0.0001
eGFR (ml/min/1.73 m^2^)	36.7 ± 25.3	50.1 ± 27.8	*P* < 0.0001
BNP/NTproBNP staging(Ⅰ/Ⅱ/Ⅲ/Ⅳ)	7/18/24/264	34/78/99/467	*P* < 0.0001
Liver function laboratory data			
Glutamic-oxaloacetic transferase (IU/L)	36.6 ± 50.7	31.8 ± 35.1	*P* = 0.0361
Glutamic-pyruvic transferase (IU/L)	28.4 ± 36.9	29.8 ± 46.5	*P* = 0.7593
Lactate dehydrogenase (U/L)	266.2 ± 129.8	242.4 ± 115.9	*P* = 0.0014
Laboratory data related to nutrition			
Albumin (g/dl)	3.5 ± 0.6	3.7 ± 0.6	*P* < 0.0001
Total cholesterol (mg/dl)	162.5 ± 40.9	171.3 ± 40.7	*P* = 0.0019
Low-density lipoprotein (mg/dl)	94.1 ± 36.4	100.8 ± 36.5	*P* = 0.0080
Serum cholinesterase (U/L)	216.3 ± 82.5	258.5 ± 84.6	*P* < 0.0001
Malnutrition parameter			
GNRI (Geriatric nutrition risk index)	94.2 ± 13.5	98.8 ± 13.7	*P* < 0.0001

Values are shown as means ± one standard deviation; ICD, implantable cardioverter-defibrillator; CRT, cardiac resynchronization therapy; eGFR, estimated glomerular filtration rate; NYHA, New York Heart Association Classification; ns, no significance.

$\begin{array}{cc} \mathrm{GNRI}(\mathrm{Geriatric}\;\mathrm{Nutrition}\;\mathrm{Risk}\;\mathrm{Index})\; & =14.89\times Alb+41.7\times (\mathrm{current}\;\mathrm{body}\;\mathrm{weight})/(\mathrm{standard}\;\mathrm{body}\;\mathrm{weight})(\mathrm{non}\text{-}\mathrm{HF}\;\mathrm{patient}).\;\\ \; & =14.89\times Alb+41.7\times (\mathrm{dry}\;\mathrm{body}\;\mathrm{weight})/(\mathrm{standard}\;\mathrm{body}\;\mathrm{weight})(\mathrm{HD}\;\mathrm{patient}).\; \end{array}$

**Table 2:** Comparison of medications and two-dimensional echocardiographic parameters and scintigraphic parameters in patients with and those without cardiac events.

	Cardiac event group (*n* = 313)	Non-cardiac event group (*n* = 678)	*P*-value
Medications			
ACE/ARBs	185 (59.1%)	392 (57.8%)	ns
*β*-blockers	288 (92.0%)	628 (92.6%)	ns
Loop diuretics	214 (68.3%)	536 (79.0%)	ns
Anti-aldosterone agents	104 (33.2%)	294 (43.3%)	ns
Anti-vasopresin agents	90 (28.7%)	178 (26.2%)	ns
Amiodarone	129 (41.2%)	170 (25.1%)	*P* < 0.0001
Statins	108 (34.5%)	69 (10.1%)	*P* = 0.0002
Findings of echocardiography			
M-mode			
LVDd (mm)	56.7 ± 11.4	55.8 ± 8.7	ns
LVDs (mm)	47.8 ± 12.5	46.2 ± 10.1	*P* = 0.0314
LAD (mm)	44.5 ± 8.1	43.3 ± 8.1	*P* = 0.0224
Modified Simpson method			
LVEF (%)	32.5 ± 11.9	32.7 ± 11.0	ns
LVEF < 40%/LVEF≧40%	215/98	487/191	ns
EDV (ml)	164.8 ± 78.4	150.5 ± 55.9	*P* = 0.0013
ESV (ml)	115.5 ± 71.7	102.7 ± 50.9	*P* = 0.0017
Tissue Doppler method			
Septal *E*/*e*′	19.7 ± 8.3	17.1 ± 7.2	*P* < 0.0001
Septal *E*/*e*′ > 15/Septal *E*/*e*′ ≦ 15	221/92	372/306	*P *< 0.0001
Cardiac MIBG parameters			
Washout ratio (%)	33.2 ± 15.1	25.3 ± 13.6	*P* = 0.0223
Early standardized HMR	2.05 ± 0.46	2.23 ± 0.45	*P* < 0.0001
Late standardized HMR	1.72 ± 0.41	2.00 ± 0.48	*P* < 0.0001

Values are shown as means ± one standard deviation; ACE-I, angiotensin-converting enzyme inhibitors; ARB, angiotensin receptor blockers; LAD, left atrial diameter; LV, left ventricular; LVEF, left ventricular ejection fraction; LVDd, end-systolic left ventricular diameter; IVSTd, end-diastolic interventricular septal wall thickness; PWTd, end-diastolic posterior wall thickness; EDV, left ventricular end-diastolic volume; ESV, left ventricular end-systolic volume; Dct, left ventricular deceleration time; ns, no significance, HMR, heart-to-mediastinum ratio.

In the multivariate logistic regression analysis excluding serum ChE (model 1), significant variables were NYHA functional class (OR: 10.1, *χ*^2^ = 67.1, *P* < 0.0001), ESV (OR: 1.002, *χ*^2^ = 4.74, *P* = 0.0295), eGFR (OR: 0.985, *χ*^2^ = 23.6, *P* < 0.0001) and late sHMR (OR: 0.333, *χ*^2^ = 38.7, *P* < 0.0001). The ROC AUC of model 1 was 0.751. In the logistic regression analysis with serum ChE (model 2), significant variables were NYHA functional class (OR: 14.3, *χ*^2^ = 48.7, *P* < 0.0001), ESV (OR: 1.003, *χ*^2^ = 6.15, *P* = 0.0131), eGFR (OR: 0.987, *χ*^2^ = 16.4, *P* < 0.0001), late sHMR (OR: 0.346, *χ*^2^ = 35.1, *P* < 0.0001), and serum ChE (OR: 0.996, *χ*^2^ = 10.72, *P* = 0.0011) (Table 3). The ROC-AUC of model 2 was 0.814. Of the two curves, the model including serum ChE had a significantly larger AUC (*P* < 0.0001).

**Table 3:** Results of logistic multivariate analyses.

Variable	Multivariate analysis				
		95% CI			
	Odds ratio	Lower	Upper	*P*-value	Reciprocal
NYHA functional class	14.3	7.843	28.36	<0.0001	0.069
ESV	1.003	1.0007	1.006	0.0131	0.996
eGFR	0.987	1.001	1.007	<0.0001	1.012
Late standardized HMR	0.346	0.239	0.495	<0.0001	2.887
Serum cholinesterase	0.996	0.994	0.999	0.0011	1.003

Net reclassification analysis was performed using the four-parameter logistic model created by the combination of NYHA functional class, ESV, eGFR and late sHMR (model 1) and by the five-parameter model that included ChE (model 2) [Table 4A]. Of the 314 patients who died of a cardiac event, classification was improved in 22 patients and was made worse in 6 patients using the five-parameter model. The net gain in reclassification proportion was 5.4% (*P* = 0.0015). Of the 677 patients who did not die of a cardiac event, 14 were reclassified upward and 248 patients were reclassified downward, with a net gain in reclassification of −57.4% (*P* < 0.0001). The NRI in all subjects was 62.7% (*P* < 0.0001) [Table 4B]. Thus, model 2 significantly improved the identification of patients at low risk of cardiac death when compared to model 1. Based on logistic regression analysis, an equation with five variables was used to estimate the prediction of CEs (model 2):

$$\begin{array}{rl} & f(\mathrm{NYHA}\;\mathrm{functional}\;\mathrm{class},\;\mathrm{ESV},\;\mathrm{eGFR},\;\mathrm{late}\;\mathrm{sHMR},\;\mathrm{ChE})=\\ & \;1/(1+\mathrm{Exp}[-(b_{\mathrm{intercept}}+b_{\mathrm{NYHA}*}\mathrm{NYHA}\;\mathrm{Class}+b_{\mathrm{ESV}*}\mathrm{ESV}\\ & \;+b_{\mathrm{eGFR}}*\mathrm{eGFR}+b_{\mathrm{late}\;\mathrm{sHMR}}*\mathrm{late}\;\mathrm{sHMR})+b_{\mathrm{ChE}}*\mathrm{ChE}])\\ & \;*100(*\mathrm{asterisk}\;\mathrm{denoting}\;\mathrm{multiplication}), \end{array}$$

where the variables *b*_intercep_, *b*_NYHA_, *b*_ESV_, *b*_eGFR_, *b*_late sHMR_, and *b*_ChE_ derived from the parameter estimates of the model were 1.875, 2.655, 0.003, −0.012, −1.060, and −0.003 (*χ*^2^ = 233.3, *P* < 0.0001), respectively.

**Table 4A:** Reclassification analysis in patients who died and those who did not die of a cardiac event.

Model 1 (NYHA + ESV + eGFR + late sHMR)	Model 2 (NYHA + ESV + eGFR + late sHMR + serum cholinesterase)		
	≦5%	5< and ≦25%	25%<
Subjects who died			
≦5%	1	0	0
5< and ≦25%	0	58	22
25%<	0	6	227
Subjects who survived			
≦5%	0	14	0
5< and ≦25%	0	403	0
25%<	0	248	12

**Table 4B:** Net reclassification in patients who died and those who did not die of a cardiac event.

	Improved	Made worse	Same	NRI (%)	*P*-value
Subjects who died	22	6	286	5.4	*P* = 0.0015
Subjects who survived	248	14	415	−57.3	<0.0001
Entire population				62.7	<0.0001

In the present formula, NYHA functional class (class Ⅰ/1, class Ⅱ/2, class Ⅲ/3, class Ⅳ/4) was a categorical variable, and ESV, eGFR, late sHMR and ChE were continuous variables.

Among the significant univariate variables (Table 5), serum ChE, eGFR and late sHMR were identified as significant independent prognostic determinants in multivariate Cox analysis together with NYHA functional class, ESV and amiodarone use, with hazard ratios and 95% CIs of 0.998, 0.996–0.999 (*P* = 0.0034), 0.994, 0.988–0.999 (*P* = 0.0250) and 0.532, 0.398–0.709 (*P* < 0.0001), respectively.

**Table 5:** Results of univariate and multivariate analyses.

	Univariate analysis					Multivariate cox proportional-hazards model analysis				
			95% CI					95% CI		
	*χ* ^2^	Hazard ratio	Lower	Upper	*P*-value	*χ* ^2^	Hazard ratio	Lower	Upper	*P*-value
Age	55.9	1.037	1.027	1.047	<0.0001	11.6	1.021	1.008	1.034	0.0006
NYHA functional class	22.0	2.491	2.243	2.758	<0.0001	90.4	2.044	1.731	2.251	<0.0001
VT/Vf	26.5	2.013	1.558	2.575	<0.0001	3.80	1.286	0.998	1.503	0.0511
Hemoglobin	30.7	0.870	0.828	0.913	<0.0001	2.46	0.959	0.888	1.013	0.1161
Estimated GFR	58.5	0.983	0.979	0.998	<0.0001	5.02	0.994	0.988	0.999	0.0250
BNP/NTproBNP staging	29.1	1.536	1.299	1.846	<0.0001	0.07	1.074	0.850	1.265	0.7778
Amiodarone	36.5	2.053	1.634	2.571	<0.0001	14.1	1.756	1.283	2.189	0.0002
Statins	7.73	0.721	0.569	0.909	0.0054	0.03	1.049	0.786	1.331	0.8486
ESV	16.3	1.004	1.001	1.005	<0.0001	5.16	1.003	1.001	1.005	0.0230
Septal *E*/*e*' > 15 or Septal *E*/*e* ≦ 15	16.8	1.692	1.311	2.204	<0.0001	2.97	1.253	0.971	1.638	0.0847
Serum cholinesterase	65.9	0.994	0.992	0.995	<0.0001	8.60	0.998	0.996	0.999	0.0034
Late standardized HMR	66.9	0.356	0.275	0.458	<0.0001	18.6	0.532	0.398	0.709	<0.0001

**Figure 2:**
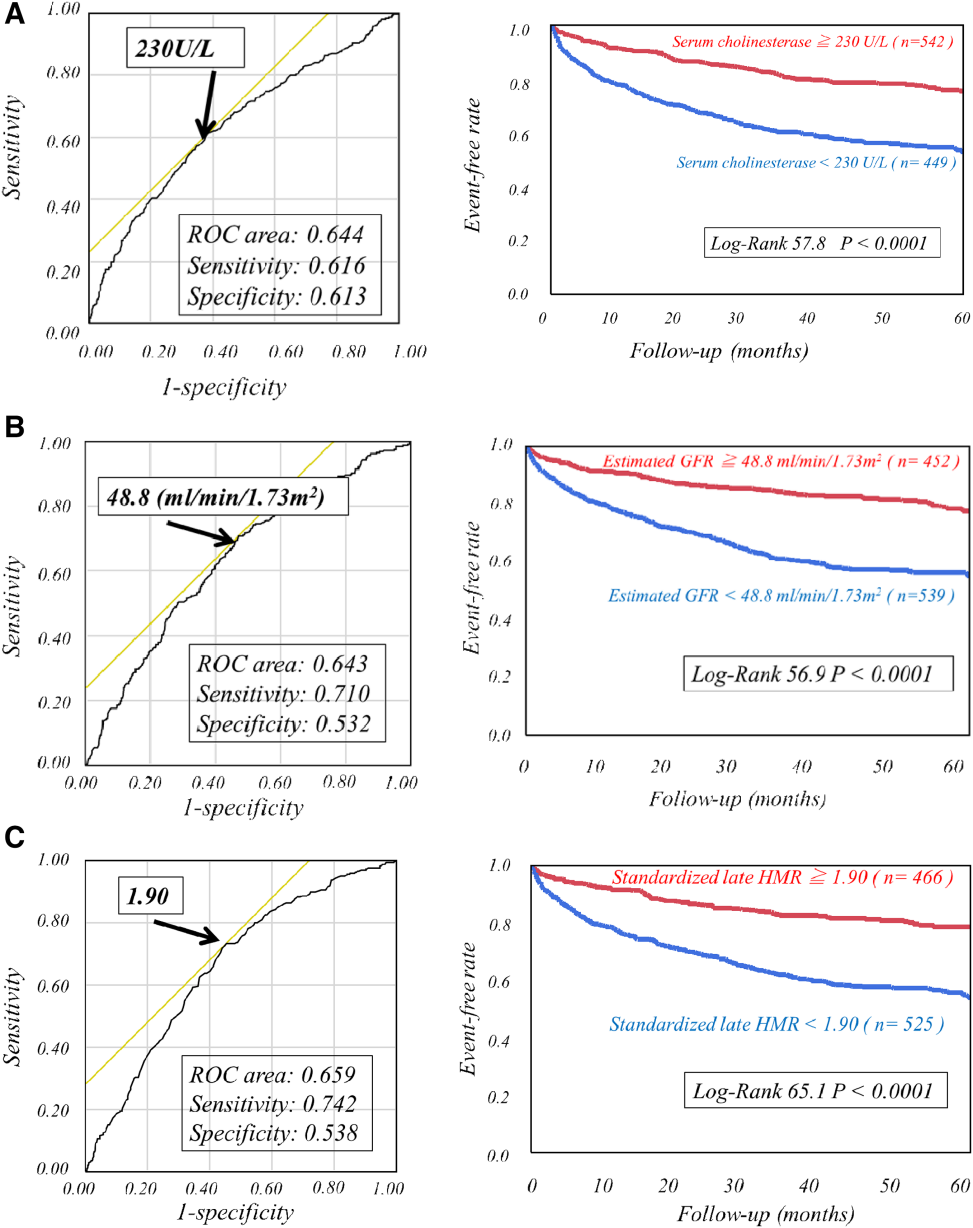
Kaplan-Meier event-free curves with ROC analysis cutoff values of serum cholinesterase of 230 U/L (**A**), estimated glomerular filtration rate of 48.8 ml/min/1.73 m^2^ (**B**), and late standardized MIBG heart-to-mediastinum ratio (sHMR) of 1.90 (**C**) clearly identified high-risk patients from low-risk patients.

**Figure 3:**
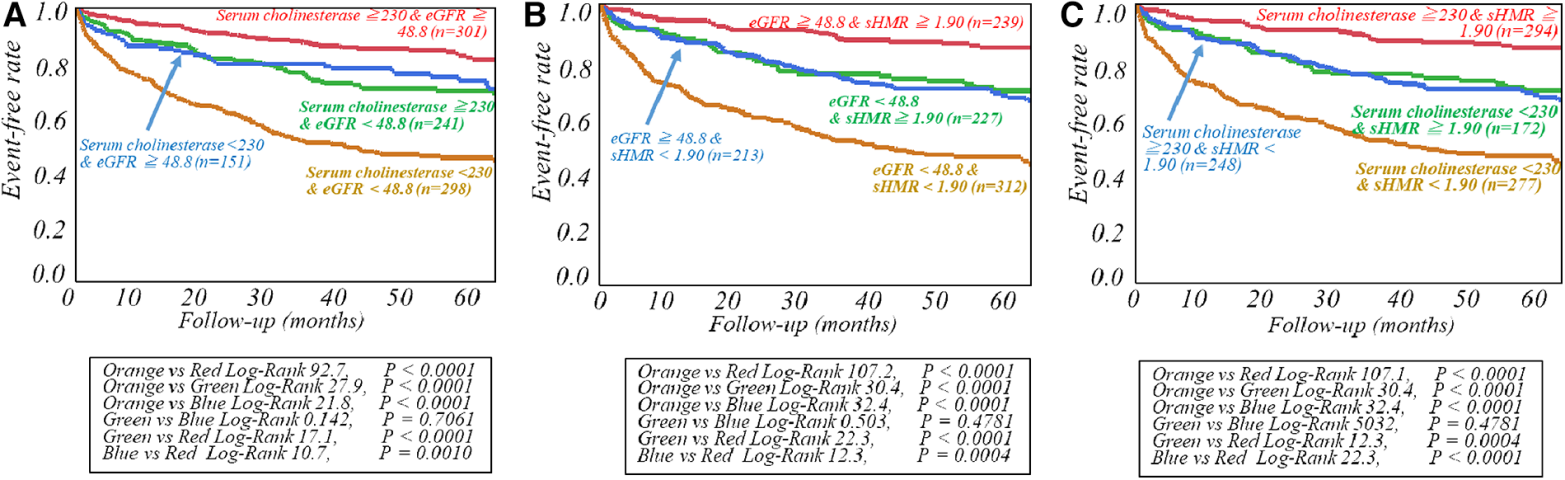
Kaplan-Meier event-free curves generated with two of the three prognostic variables, including serum cholinesterase, estimated glomerular filtration rate (eGFR), and late standardized MIBG heart-to-mediastinum ratio (sHMR), were used to clearly classify patients into low-, intermediate-, and high-risk groups. Risk stratification of heart failure patients was performed by combining serum cholinesterase and estimated glomerular filtration rate (eGFR) in A, estimated glomerular filtration rate (eGFR) and late standardized MIBG heart-to-mediastinum ratio (sHMR)in B and serum cholinesterase and late standardized MIBG heart-to-mediastinum ratio (sHMR)in C, respectively.

**Figure 4:**
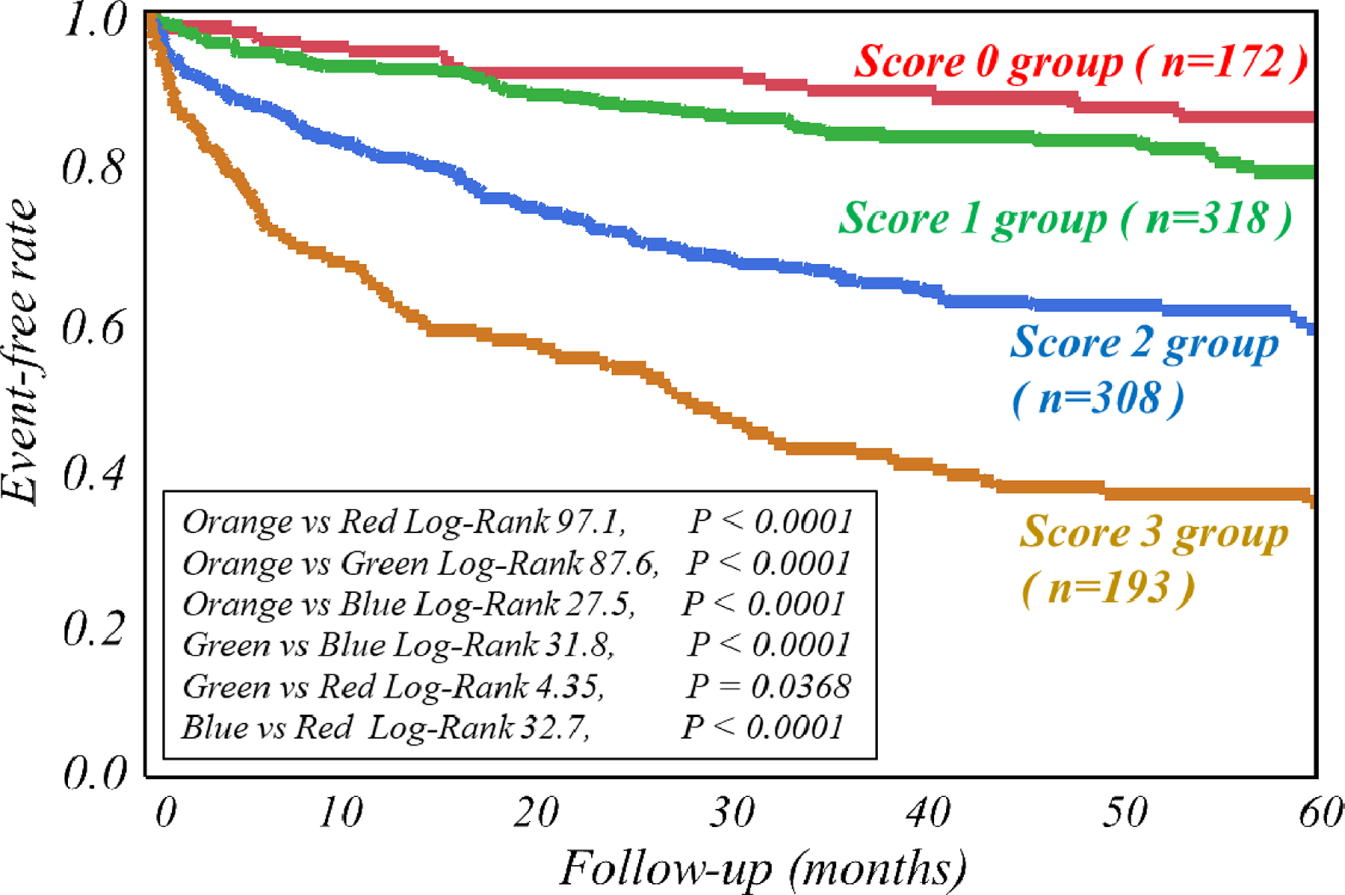
Kaplan-Meier event-free curves clearly show that accumulation of the three abnormal parameters (i.e., serum cholinesterase <230 U/L, estimated glomerular filtration rate (eGFR) < 48.8 ml/min/1.73 m^2^ and late standardized heart-to-mediastinum ratio of MIBG activity (sHMR) < 1.90) synergistically decreased event-free rates. The score category (0–3) indicates the number of the three abnormal parameters.

The respective cut-off values for serum ChE, eGFR and late sHMR were calculated from the ROC curve. By using cut-off values of the three parameters, patients were clearly categorized into high-risk and low-risk sub-groups (Figure 2). Patients who had a serum ChE level of less than 230 U/L, eGFR of less than 48.8 ml/min/1.73 m^2^ or late sHMR of less than 1.90 had significantly lower cardiac event-free rates than did their counterparts (Figure 2). The combination of two of the three parameters (ChE, eGFR and late sHMR) more clearly discriminated HF patients in three risk categories: low, intermediate, and high-risk groups (Figure 3). Further accumulation of the three parameters (i.e., serum ChE < 230 U/L, eGFR < 48.8 ml/min/1.73 m^2^ and late sHMR < 1.90) was demonstrated to increase CE risks in HF patients (Figure 4).

## Discussion

The present study revealed a synergistic role of serum ChE as a malnutritional parameter in risk stratification of patients with systolic HF, conventionally assessed by renal dysfunction and cardiac sympathetic denervation.

### Cardiac sympathetic nerve denervation and malnutrition

A low nutritional status worsens the prognosis of HF patients (2, 17). In HF patients, impaired absorption and increased permeability due to liver dysfunction, intestinal edema and decreased appetite due to right HF are possible causes of a malnutritional state. In elderly HF patients, furthermore, inadequate energy intake, increased energy expenditure, and impaired anabolism can combine to form a malnutritional state that is prone to water retention and infection. In addition, the parasympathetic nervous system is predominantly involved in the secretion of digestive enzymes such as glucosidase, peptidase, and lipase, and digestion and absorption are inhibited in a hyper-sympathetic state, which likely contributes to malnutrition in HF patients. In the present study, levels of liver enzymes were also significantly higher in the CE group than in the non-CE group. Serum ChE level, an indicator of liver dysfunction and malnutrition, was also an independent prognostic factor together with an indicator of cardiac sympathetic dysfunction. Moreover, the combination of these two parameters suggests that further risk stratification of HF patients is possible.

### Renal dysfunction and malnutrition

Recently, the concept of protein energy wasting (PEW), defined as a depletion of protein energy sources, has been used to define malnutrition in patients with renal dysfunction. The causes of PEW include inadequate nutritional intake, systemic inflammatory response syndrome due to a cytokine storm including HF, uremia, endocrine abnormalities (increased insulin resistance, overproduction of catabolic hormones, decreased production of anabolic hormones), and metabolic acidosis (18, 19).

The vicious cycle leads to hypoproteinemia and albuminemia.

In addition, hyperglycemia and increased insulin resistance in patients with renal dysfunction lead to abnormal glucose metabolism. In the liver, this leads to accelerated glycogen breakdown, increased glycogenesis, and inhibition of sugar and amino acid oxidation (18). Furthermore, for adipose tissue, renal dysfunction induces insulin-dependent suppression of glucose oxidation, increased lactate production and fatty acid release. Levels of total cholesterol, HDL and LDL are decreased (19).

In the present study, the synthesis of lipids and proteins was also suppressed in the CE group compared to that in the non-CE group, with significantly lower blood levels of lipid profile and protein/albumin in the CE group. Serum ChE level, a surrogate parameter for lipid and protein-albumin synthesis in the liver, was also significantly lower in the CE group. Serum ChE was an independent prognostic factor, as was eGFR, a surrogate parameter for renal dysfunction, suggesting that the combination of these two parameters might enable synergistic risk stratification of HF patients.

### Cardiac sympathetic nerve denervation and renal dysfunction

When hypoperfusion is sustained due to HF, the renin-angiotensin-aldosterone system, cardiac sympathetic nerve function, arginine vasopressin, endothelin I, and atrial natriuretic peptide are constantly activated (8). Long-lasting HF leads to increased cardiac sympathetic function, contraction of import and export arteries, and decreased GFR, which further contribute to renal dysfunction.

Decreased GFR leads to anemia due to impaired erythropoietin production and other causes. Anemia worsens heart failure, increases cardiac sympathetic function and leads to refractory HF (8, 20).

This study also showed significant cardiac sympathetic dysfunction and renal dysfunction in the CE group compared to those in the non-CE group. Moreover, cardiac sympathetic denervation and renal dysfunction were selected as independent prognostic factors, indicating that the combination of these two parameters might enable clearer risk stratification in HF patients.

## Limitations

The present study was designed as an observational and non-interventional cohort study of patients with irreversible systolic HF at a single facility.

A large multicenter intervention study based on the results of our study could contribute to the development of better prevention and treatment strategies, including diet and cardiac rehabilitation, using appropriate indications for malnutrition in HF patients with comorbid cardiac cachexia who are at high risk of death. In the present study, there was no evaluation of the Prognostic Nutrition Index (PNI) or Controlling Nutritional Status (CONUT), which have been reported to have a prognostic value for malnutrition in HF patients (21, 22).

In HF patients at risk for nutritional impairment, approaches to improving nutritional status have been reaffirmed as part of the role of cardiac rehabilitation. Improvement of nutritional status is expected to be effective for improving exercise capacity. It has also been reported that cardiac rehabilitation increases muscle mass, prevents muscle hypercatabolism, and improves nutritional status (23, 24).

There are two main types of therapy for HF patients: pharmacological and non-pharmacological. The use of percutaneous coronary intervention, catheter ablation, ICDs and biventricular pacemakers, transcatheter aortic valve implantation, MitraClip ® implantation, and other non-pharmacologic therapies have received attention, and the importance of nutritional management, including diet and exercise rehabilitation, should be further discussed.

## Conclusion

A lower level of serum ChE can independently and synergistically predict the risk of cardiac mortality in systolic HF patients with cardiac sympathetic innervation and renal dysfunction.

## Data Availability

The raw data supporting the conclusions of this article will be made available by the authors, without undue reservation.
